# PPAR**γ** Promotes Growth and Invasion of Thyroid Cancer Cells

**DOI:** 10.1155/2011/171765

**Published:** 2011-12-12

**Authors:** William M. Wood, Vibha Sharma, Kevin T. Bauerle, Laura A. Pike, Qiong Zhou, Deborah L. Fretwell, Rebecca E. Schweppe, Bryan R. Haugen

**Affiliations:** ^1^Division of Endocrinology, Metabolism and Diabetes, Department of Medicine, University of Colorado Denver, Aurora, CO 80045, USA; ^2^University of Colorado Cancer Center, University of Colorado Denver, Aurora, CO 80045, USA

## Abstract

Undifferentiated (anaplastic) thyroid cancer (ATC) is one of the most aggressive human malignancies and no effective therapy is currently available. We show here that PPAR**γ** levels are elevated in cells derived from ATC. Depletion of PPAR**γ** in HTh74 ATC cells resulted in decreased cell growth, cell cycle arrest and a reduction in pRb and cyclin A and B1 levels. We further showed that both flank and orthotopic thyroid tumors derived from PPAR**γ**-depleted cells grew more slowly than PPAR**γ**-expressing cells. When PPAR**γ** was overexpressed in more differentiated thyroid cancer BCPAP cells which lack PPAR**γ**, there was increased growth and raised pRb and cyclin A and B1 levels. Finally, PPAR**γ** depletion in ATC cells decreased their invasive capacity whereas overexpression in PTC cells increased invasiveness. These data suggest that PPAR**γ** may play a detrimental role in thyroid cancer and that targeting it therapeutically may lead to improved treatment of advanced thyroid cancer.

## 1. Introduction

Thyroid cancer is the most common endocrine malignancy with 44,000 new cases each year and approximately 400,000 Americans are currently living with the disease. While many patients diagnosed with thyroid cancer do very well after standard therapy (surgery, radioiodine, levothyroxine replacement), approximately 1,700 patients with poorly differentiated thyroid cancer die each year and many others suffer from progressive, symptomatic disease. Furthermore, anaplastic thyroid cancer is one of the most lethal cancers, with a 50% survival of only 6 months. A better understanding of how thyroid cancer progresses from differentiated to undifferentiated cancer will help us to develop critical markers of this disease progression and novel therapies to treat patients with advanced thyroid cancer.

Peroxisome proliferator-activated receptors (PPARs) are members of the nuclear hormone receptor superfamily which are ligand-dependent transcription factors that regulate many important physiological processes [1]. PPARs exist as three different isoforms (*α*, *γ*, and *δ*) that play major roles in adipose tissue development and lipid metabolism [2, 3]. In addition to its widely understood role in adipocyte biology, the PPAR*γ* isoform has been implicated in regulating carcinogenesis [4]. Furthermore, PPAR*γ* activators of the thiazolidinedione class (TZDs) such as rosiglitazone have been reported to slow the growth of colon [5] and lung [6, 7] tumors. However, the role of PPAR*γ* in tumorigenesis is controversial, stemming from the discrepancy between the anticancer effects suggested by *in vitro* studies, and the tumor-promoting capacity reported in mouse models of colon cancer [8]. This could be a consequence of the fact that cells in culture are not subjected to the microenvironment interactions necessary for complex tumor formation *in vivo*. In addition, many *in vitro* studies reveal that the antiproliferative effects are seen only when concentrations of PPAR*γ* agonist greatly exceed that needed to saturate the receptor. Growth inhibition by PPAR*γ* ligands has also been reported in cells that do not express PPAR*γ*, questioning a legitimate role for the receptor in mediating this effect [9]. A recent article by Wei et al. [10] reviews these “off-target” mechanisms that underlie the antitumor activity of TZDs specifically and provide evidence that, relative to tumor cells, nonmalignant cells are resistant to these PPAR*γ*-independent antitumor effects. Thus, the anticancer action attributed to PPAR*γ* ligands may not be through classical PPAR*γ* signaling. There is an extensive literature on the effect of PPAR*γ* ligands on growth of thyroid cancer cells *in vitro* and in mouse xenografted tumors [11–15]. It is not clear if the effects of PPAR*γ* agonists are receptor-dependent or independent [9]. Furthermore, we have discovered that many of the cell lines used in these studies were not of thyroid origin [16]. In fact, one of the most responsive cell lines, which expresses PPAR*γ*, was derived from a melanoma [17, 18]. Finally, clinical trials using PPAR*γ* agonists in advanced thyroid cancer have been disappointing and PPAR*γ* levels were not assessed in most tumors [19, 20]. Clearly, a better understanding of the role of PPAR*γ* in advanced thyroid cancer is needed.

## 2. Materials and Methods

### 2.1. Cell Lines and Chemicals

Cell lines were obtained from the primary source or the American Type Culture Collection (ATCC) with the exception of the following. BCPAP cells were kindly provided by Dr. M. Santoro (Medical School, University of Naples Federico II, Naples, Italy). K1 cell lines were provided by Dr. Wynford-Thomas (Cardiff University, Cardiff, UK). C643 and HTh74 cells were from Dr. K. Ain (University of Kentucky, Lexington, KY) with permission from Dr. N. E. Heldin (University Hospital, Uppsala, Sweden), and the TPC1 cells were kindly provided by Dr. S. Jhiang (Ohio State University, Columbus, OH). The cell lines used in this study were analyzed by short tandem repeat profiling and shown to be unique [16]. Cells were grown in RPMI (Invitrogen, Carlsbad, CA) containing 5% FBS (Hy-Clone Laboratories, Logan, UT) and maintained at 37°C in 5% CO_2_. Rosiglitazone (thiazolidinedione, TZD) was provided by GlaxoSmithKline.

### 2.2. Viable Cell Proliferation Assays

Cells were plated in duplicate in 6 cm dishes in RPMI containing 5% FBS at 45,000 cells/dish. The medium containing 1 or 10 *μ*M Rosi, or DMSO was replaced every 3 days for 6 days. Cells in the medium were collected, and adherent cells were harvested using Trypsin-EDTA at the time indicated, quenched with RPMI containing 5% serum, and resuspended in 0.5 mL PBS. Viable cells were counted using a Beckman Coulter ViCell cell counter, which counts 100 fields of cells and calculates viable cells using trypan blue exclusion.

### 2.3. Cell Cycle Analysis

Cells were plated in 6 cm dishes at 1 × 10^5^/well in RPMI containing 0.1% FBS. After 24 h at 37°C to restrict cells to G0, the medium was replaced with RPMI supplemented with 5% FBS. After a further 24 h, cells were collected by trypsinization and washed twice in ice-cold PBS. Cell pellets were resuspended in a saponin/propidium iodide solution (0.3% saponin, 25 mg/mL propidium iodide, 0.1 mM EDTA, and 10 *μ*g/mL RNase A) and incubated at 37°C for 24 h. Cell cycle distribution was determined by flow cytometry using a Beckman Coulter FC500 at the University of Colorado Cancer Center Flow Cytometry Core. ModFit LT (Verity Software House, Topsham, ME) was used for cell cycle modeling and doublet discrimination.

### 2.4. Western Blot Analysis

Nuclear protein was prepared from cells using a nuclear extract kit supplied by Active Motif (catalog number 400100, Carlsbad, CA). For preparation of whole cell extracts, cells were trypsinized, centrifuged at 1000 rpm for 5 min, and suspended in extraction buffer (EB; 1% Triton X-100, 10 mM Tris HCL, pH 7.4, 5 mM EDTA, 50 mM NaF, 1 mM phenylmethylsulfonyl fluoride, and 2 mM Na_3_VO_4_ supplemented with 1x complete protease inhibitors (Roche Diagnostics)). Cell debris was removed by centrifugation at 13,000 rpm for 10 min at 4°C. Protein concentrations were determined using the DC protein assay (Bio-Rad Laboratories, Hercules, CA). Diluted samples containing equal amounts of protein (60 *μ*g) were mixed with 2x Laemmli sample buffer (Bio-Rad Laboratories). Proteins were separated on a 10% SDS-polyacrylamide gel and transferred to a polyvinylidene difluoride membrane (PVD). The membrane was blocked with 1x TBST (20 mmol/L Tris-HCl (pH 7.6), 8.5% NaCl, and 0.1% Tween 20) containing 5% nonfat dry milk at room temperature for 2 h and incubated in the appropriate primary antibody in 1x TBST containing 5% nonfat dry milk at 4°C overnight. The source of primary antibodies was as follows: from Santa Cruz Biotechnology, Santa Cruz, CA, PPAR*γ* (rabbit polyclonal, sc-7196), PPAR*δ* (*β*) (rabbit polyclonal, sc-7197), cyclin A (rabbit polyclonal, sc-596), cyclin B1 (rabbit polyclonal, sc-752), cyclin D1 (mouse monoclonal, sc-20044), cyclin D2 (rabbit polyclonal, sc-593), and cdc2/cdk1 (rabbit polyclonal, sc 8395); from Cell Signaling Technology, MA, phosphoRb (Ser 807/811) (rabbit monoclonal, 9308S), p21 (rabbit monoclonal, 2947); p27 was from Novus Biologicals (100–1949). Poly (ADPribose) polymerase (PARP-rabbit polyclonal, AA16661, Millipore) and *β*-actin (mouse monoclonal, A-5441, Sigma) were used as loading controls for nuclear and whole cell extracts, respectively. After washing with 1xTBST, membranes were incubated for one hour at room temperature with either donkey-anti-rabbit or sheep-anti-mouse IgG conjugated to horse-radish peroxidase (HRP) (GE Healthcare, UK) at a dilution of 1 : 1000. SuperSignal West Pico Chemiluminescent Substrate, an enhanced HRP detection reagent from Thermo Scientific (Rockford, IL), was used for immunodetection. Blots were reprobed following stripping with Re-Blot Plus Strong (Millipore).

### 2.5. Quantitative Reverse Transcription-PCR (qRT-PCR)

Total RNA was isolated from shPPAR*γ* or scrambled HTh74 cells using the RNeasy Mini Kit (Quiagen, Valencia, CA) as per the manufacturer's protocol. The mRNA for PPAR*γ* was measured by real-time quantitative RT-PCR using ABI PRISM7700. The sequences of forward and reverse primers as designed by Primer Express (PE ABI) were 5′-AGT GGA GAC CGC CCA GGT-3′ and 5′-GGG CTT GTA GCA GGT TGT CTT G-3′. The TaqMan fluorogenic probe used was 6FAM-TGC TGA ATG TGA AGC CCA TTG AAG ACA-TAMRA. Amplification reactions, thermal cycling conditions, and generation of a standard curve have been described previously [18].

### 2.6. PPAR*γ* shRNA Knockdown

We used a lentiviral mediated shRNA system from Sigma (St. Louis, MO) and followed the manufacturer's protocol. Lentiviral particles contain shRNA toward PPAR*γ*-specific sequences as well as a scrambled (scr) sequence that consists of 5 nucleotides that do not match any known gene transcript in both the murine and human genome. The transduced cells are selected by puromycin resistance and then assessed for correct insertion/RNA inhibition by qRT-PCR or western blot for PPAR*γ*. The concentration of puromycin used to select for DNA construct incorporation cells was 0.5 *μ*g/mL.

### 2.7. PPAR*γ* Overexpression

A plasmid containing the coding region of mouse PPAR*γ* (pCMX-PPAR*γ*) and pQCXIP retroviral expression vector were kindly provided by Dr. L. Jameson (Northwestern University, Chicago) and Dr. S. Nordeen (University of Colorado Denver, Anschutz Medical Campus), respectively. The plasmid pCL-Ampho and the BOSC cell line, a derivative of the HEK-293 cell line, were provided by Dr. H. Ford (University of Colorado Denver, Anschutz Medical Campus). The PPAR*γ*-coding region was PCR amplified from pCMX-PPAR*γ* using primers with terminally engineered Not I and Age I restriction sites and, after shuttling through PCR 2.1, the excised Not I/Age I fragment was gel-purified and directionally inserted into Not I/Age digested pQCXIP to generate the pQCXIP-PPAR*γ* retroviral expression vector. The insert was sequenced in its entirety and no errors were found.

Virus was produced by transfection (Effectene; Qiagen) of BOSC cells with pQCXIP-PPAR*γ* or pQCXIP alone in combination with pCL-Ampho, according to manufacturer's instructions. Briefly, BOSC cells were seeded in 10 cm dishes to be 60–70% confluent the next day. The vectors pCL-Ampho (2 *μ*g) and pQCXIP or pQCXIP-PPAR*γ* (4 *μ*g) were mixed with EC buffer (final volume of 300 *μ*L). Following the addition of 16 *μ*L enhancer and 60 *μ*L effectene, the mixture was incubated at room temperature for 5 minutes and 15 minutes, respectively. The transfection mixture was then combined with 3 mL of BOSC medium and added to BOSC cells, to which 7 mL of fresh BOSC medium had been added. Media containing viral particles was collected 48, 72, and 96 hours after transfection, and each collection was aliquoted and snap-frozen.

BCPAP cells were virally transduced to generate the BCPAP-empty vector, and BCPAP-pQCXIP-PPAR*γ* sublines. Three rounds of transductions were carried out to generate each subline. BCPAP cells were seeded in 10 cm dishes to be 50–70% confluent the next day. For rounds 1 and 2, viral supernatant was mixed with growth media (RPMI supplemented with 10% FBS) at a 1 : 1 ratio (4 mls/10 cm plate; 8 *μ*g/mL polybrene) and incubated with cells for 4 hours at 37°C and 5% CO_2_. For round 3, viral supernatant was mixed with growth media (RPMI supplemented with 10% FBS) at a 1 : 2 ratio (6 mls/10 cm plate; 8 *μ*g/mL polybrene) and incubated with cells overnight at 37°C and 5% CO_2_. Selection of cells stably expressing the vector control or PPAR*γ* constructs was initiated 48 h later by treatment with 0.5 *μ*g/mL puromycin (Sigma). The pQCXIP vector ensures that cells resistant to puromycin maintain expression of the PPAR*γ* protein which is cloned upstream of an IRES-puromycin resistance cassette, eliminating the need for clonal selection of stable transfectants.

### 2.8. Flank Xenograft and Orthotopic Tumor Models

Athymic nude mice were purchased from National Cancer Institute (NCI—NCr-*nu/nu *01B74). All mice were male, 6-7 weeks old and weighing 15–30 grams. Mice were handled in accordance with the approval of the UCDHSC Animal Care and Use Committee. HTh74 cells, PPAR*γ* shRNA knockdown and scrambled controls, were grown in RPMI media supplemented with 5% FBS and suspended at 5 × 10^6^ cells/200 *μ*L sterile PBS. Mice were separated into groups of 12 for shRNA and 12 for scrambled controls and after they were anesthetized with an intraperitoneal injection of Avertin (0.5–0.7 cc of 32 mg/mL), 5 × 10^6^ tumor cells (200 *μ*L) were injected subcutaneously on the right flank of each mouse and 5 × 10^5^ cells (20 *μ*L) were introduced directly into the right thyroid lobe of the same mouse, using a Hamilton syringe aided by microscopic visualization; a location that better approximates the characteristics of human thyroid cancer. Mice were observed twice per week and flank tumors were monitored with electronic calipers. Postmortem-excised tumor volume, from both the flank and the thyroid, was estimated using the formula: tumor (length × width × height)/0.5236.

### 2.9. Invasion Assays

Following serum starvation overnight in medium containing 0.1% FBS, 2 × 10^5^ shPPAR*γ* or scrambled HTh74 cells or 10^5^ BCPAP cells transduced with PPAR*γ* or empty vector were plated in the upper chambers of Matrigel-coated transwell chambers (24-well, 8 *μ*M pore size; BD Biosciences) in 0.35 mL of RPMI medium supplemented with 0.1% FBS. Cells were allowed to invade toward 1 mL of RPMI containing 10% FBS added to the lower chamber for 24 h (BCPAP) or 48 h (HTh74). Noninvading cells on the top chamber were removed by scraping with a cotton swab, and invading cells on the lower surface were fixed with 100% methanol and stained with 3 *μ*g/mL 4′,6-diamidino-2-phenylindole (DAPI; Invitrogen). Invasive capacity of the cells was quantitated by counting DAPI-stained nuclei in five microscopic fields under 10x magnification using Metamorph software attached to a Nikon microscope.

## 3. Results

### 3.1. PPAR*γ* Activity and Expression in Authenticated Thyroid Cancer Cells: ATC Cells Overexpress PPAR*γ*

**Figure 1:**
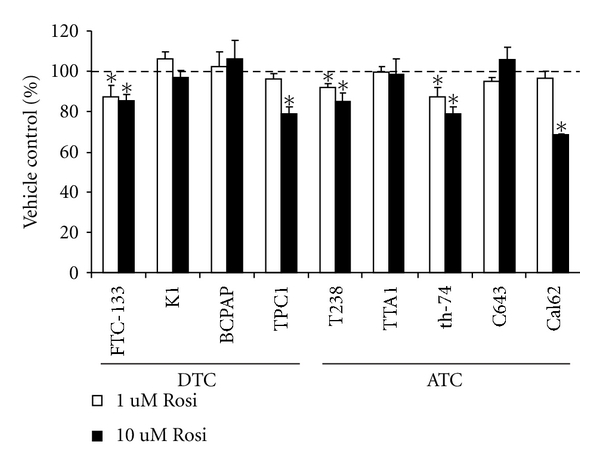
Effect of a PPAR*γ* agonist on *in vitro* growth of thyroid cancer cell lines. Cells (denoted on the *x*-axis) were treated with vehicle (DMSO), or Rosi (1 *μ*M or 10 *μ*M) every 3 days for 6 days. The effect of treatment on proliferation was determined by automated viable cell counting. Data is represented as percent viable cells/mL compared to vehicle, which is denoted by the dashed line. Mean ± S.E.M. of at least 3 independent experiments performed in duplicate is reported (*, *P* < 0.05 when compared to vehicle control).

Once our group identified unique differentiated and undifferentiated thyroid cancer cell lines [16], we reexamined the growth effects of the TZD, rosiglitazone on nine authenticated thyroid cancer cell lines (four derived from differentiated and five from undifferentiated cancers).  Figure 1 shows that there was a significant, but modest, inhibition of growth with TZD in five of the cell lines, whereas four cell lines were completely resistant to high-dose TZD. Four of the five cell lines required a very high concentration (10 *μ*M) to elicit an inhibitory effect.

**Figure 2:**
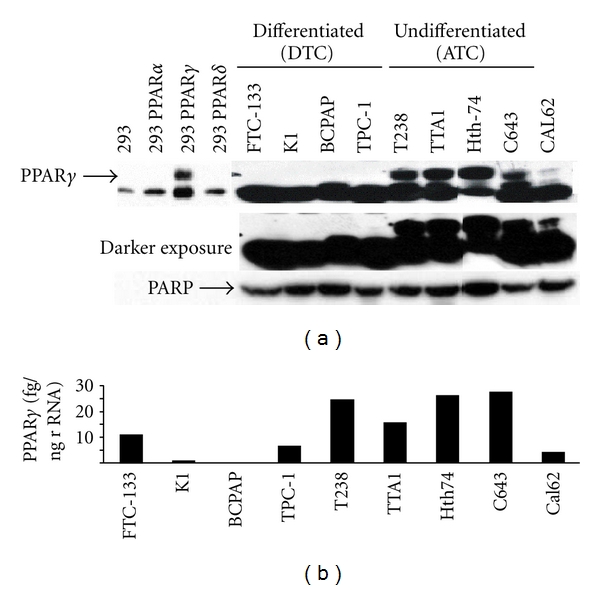
Protein and mRNA levels of PPAR*γ* in thyroid cancer cells. (a) 60 *μ*g of nuclear extract protein from various papillary and anaplastic thyroid cancer cells and 293 cells overexpressing PPAR*α*, *γ*, or *δ* was separated on a 10% SDS-PAGE gel and transferred to a PVD membrane.The blot was blocked with 10% nonfat milk and incubated overnight with PPAR*γ* rabbit polyclonal ab (sc-7196, Santa Cruz, CA) at a dilution of 1 : 500. Secondary antibodies were anti-rabbit IgG conjugated to horseradish peroxidase (GE Healthcare UK) at a 1 : 1000 dilution. PARP was quantitated as a loading control. (b) 200 ng of total RNA from the same thyroid cancer cells was subjected to qRT-PCR using an ABI PRISM7700 and the PPAR*γ*-specific oligos and probe described in Section 2.5. PPAR*γ* mRNA levels are shown per ng of 18 s rRNA.

To determine if growth inhibition by a PPAR*γ* agonist correlated with receptor expression, western blot analysis was performed on the thyroid cancer cell lines (Figure 2(a)). The specificity of antibodies raised against PPAR*γ* is quite variable, and we have tested many different antibodies most of which cross-react with PPAR*δ*, leading to confusing results in many studies. We therefore developed robust controls by overexpressing each receptor isoform (*α*, *γ*, and *δ*) in 293 cells which lack PPAR expression. Using specific antibodies (Santa Cruz, sc-1796), we have demonstrated that PPAR*α* and PPAR*δ* are expressed at high levels in these engineered cells, which makes these important controls for our studies. Western blot analysis of the nine thyroid cancer cell lines showed that this antibody specifically recognizes PPAR*γ* but also detects a nonspecific band migrating just below PPAR*γ* in all cells (Figure 2(a)). We found that only three of five cell lines expressing high PPAR*γ* protein levels demonstrated any growth inhibition with TZD treatment (T238, Hth74, and Cal62). Surprisingly, two of the four cell lines responsive to agonist treatment (FTC-133 and TPC1) lacked detectable PPAR*γ* even after prolonged exposure, indicating that growth inhibition by agonist did not correlate with receptor expression. The most striking finding was the correlation of PPAR*γ* levels with the cancer origin (DTC versus ATC). The undifferentiated thyroid cancer cells all had clearly detectable PPAR*γ*, while the differentiated thyroid cancer cells lacked protein expression of this nuclear receptor even after increased exposure time of the blot (Figure 2(a)). We also measured the PPAR*γ* mRNA levels in the nine cell lines by qRT-PCR (Figure 2(b)). Although the mRNA levels somewhat correlate with the PPAR*γ* protein levels, two DTC cells (FTC and TPC-1), both of which were responsive to agonist treatment (Figure 1), exhibited significant PPAR*γ* mRNA levels approaching a third to a half of that seen in the ATC cells. However, their PPAR*γ* protein levels were still undetectable despite prolonged exposure of the western blot (Figure 2(a)), suggesting that protein levels below the level of detection of our western assay are sufficient to elicit a response to agonist.

### 3.2. Depletion of PPAR*γ* in ATC Cells Diminishes the Agonist Response, Reduces Cell Proliferation, and Arrests the Cell Cycle as a Result of Changes in Expression of Specific Cell Cycle Factors

**Figure 3:**
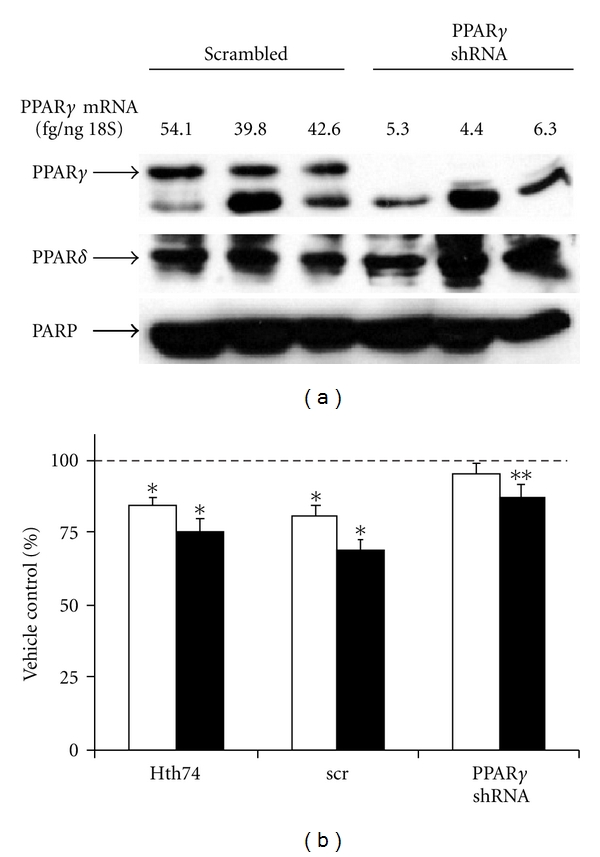
PPAR*γ* shRNA decreases PPAR*γ* mRNA and protein levels and partially abrogates growth inhibition by Rosi. (a) 40 *μ*g of nuclear extract protein from HTh74 cells transduced with either scrambled or PPAR*γ*-specific shRNA (3 independent transductions) was separated on a 10% SDS-PAGE gel, transferred to PVD, and probed with PPAR*γ* antibodies (sc-7196) as described in Figure 2(a). The blot was stripped and reprobed with antibodies to PPAR*δ* (sc-7197). PARP was quantitated as a loading control. Shown above the protein blot are PPAR*γ* RNA levels measured by qRT-PCR as in Figure 2(b). (b) Viable HTh74 cells either untransduced or transduced with scrambled (scr) or PPAR*γ* shRNA were counted after 3 days treatment with 1 *μ*M (white bars) and 10 *μ*M Rosi (black bars). The DMSO vehicle represented by the dotted line is set to 100%. Shown is the % of vehicle control for each cell type (average of 3 different expts. ± SEM. *: significantly lower than vehicle, *P* < 0.05; **: significantly lower than vehicle but higher than scr (10 *μ*M), *P* < 0.05).

**Figure 4:**
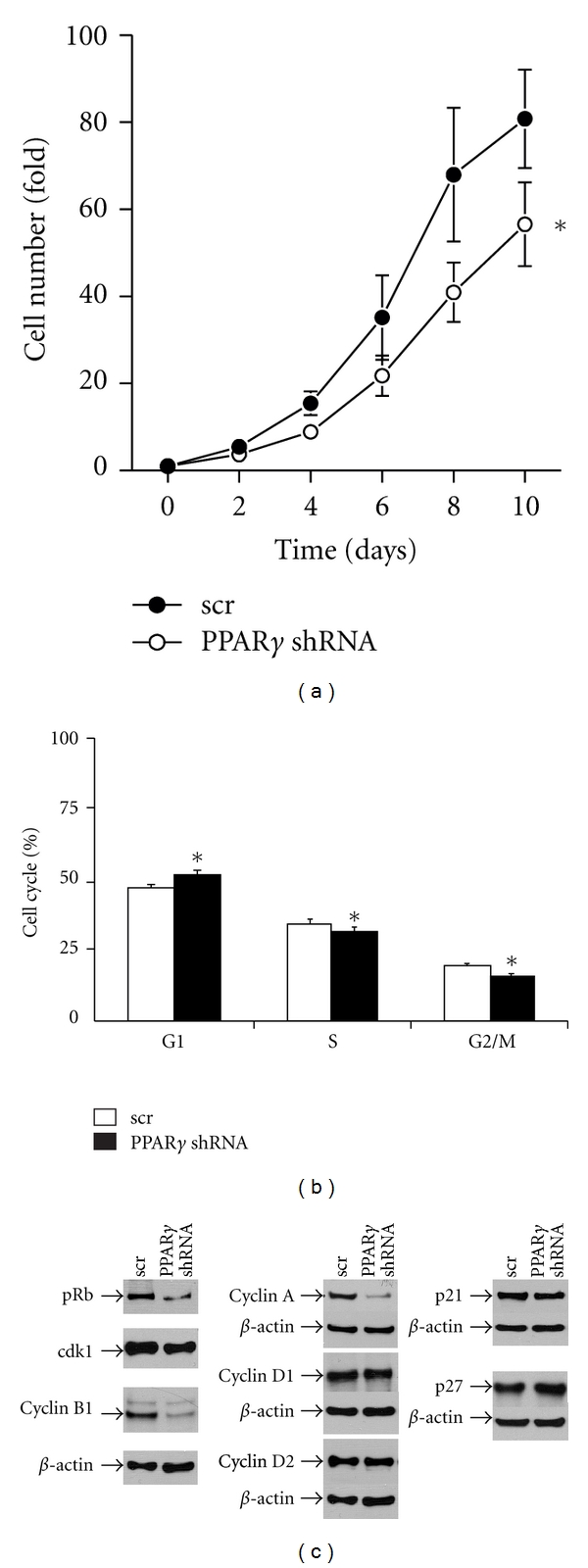
Effect of PPAR*γ* knockdown on the growth characteristics of HTh74 cells. (a) Cell growth rate was assessed by counting viable cells transduced with scrambled (scr) or PPAR*γ* shRNA at 2-day intervals. Data is represented as fold cell number over day 0 (6 expts. ± SEM, *, *P* = 0.04, 2-way ANOVA calculated over the entire growth curve). (b) scr-(white bars) and PPAR*γ* shRNA-(black bars) transduced HTh74 cells were growth arrested in 0.5% FBS for 24 h and analyzed by flow cytometry 24 h following subsequent growth in 5% FBS. Flow data (3 expts.) was quantitated for % of cells in G1, S, and G2/M phases as shown (*, G1- *P* < 0.001; S, *P* = 0.04; G2/M, *P* < 0.001: paired *t*-test). (c) 50 *μ*g of total cellular protein from HTh74 cells transduced with either scrambled or PPAR*γ*-specific shRNA was separated on a 10% SDS-PAGE gel, transferred to PVD, and probed with antibodies against the corresponding cell cycle protein whose source is described in Section 2.4 followed by the appropriate secondary antibody. *β*-actin was quantitated as a loading control.

In order to determine the contribution of PPAR*γ* to TZD response and also to examine its role in ATC versus DTC cells, we designed an shRNA strategy to knock down PPAR*γ* levels in agonist responsive HTh74 cells where it is highly expressed. Western blot analysis demonstrated complete loss of PPAR*γ* protein using a PPAR*γ*-specific shRNA when compared with scrambled shRNA control in three independent viral transductions (Figure 3(a)). We confirmed decreased mRNA by qRT-PCR verifying that loss of expression was also occurring at the mRNA level (Figure 3(a)).  Figure 3(b) demonstrates that depletion of PPAR*γ* in HTh74 ATC cells blunted, but did not completely abrogate, the modest suppression of growth by 10 *μ*M Rosi, suggesting that a high concentration of agonist has both receptor-dependent as well as receptor-independent effects on growth. Surprisingly, knockdown of PPAR*γ* alone significantly decreased growth of the ATC cells, when compared with scrambled control cells (Figure 4(a)). Flow cytometry analysis showed that this reduced growth rate could be accounted for by a modest but significant cell cycle arrest (G1 occupancy increased, S-phase and G2 /M decreased) (Figure 4(b)). A delayed G1-to-S transition is usually accompanied by a reduction in the phosphorylation state of retinoblastoma (Rb) protein which is indeed what we observed (Figure 4(c)).

To determine if the change in phosphorylation state of Rb was a result of changes in kinases, activating cyclins or inhibitors involved in its activation/deactivation, we examined the levels of several relevant cell cycle components by western blotting (Figure 4(c)). Surprisingly, instead of seeing changes in the cyclin D family, which classically activates Rb phosphorylation and G1-to-S transition, we found decreases in cyclin A, which plays a role in S-phase maintenance, and cyclin-dependent kinase 1 (cdk1) and its activating cyclin (B1) which are both associated with G2 /M function. Two previous reports using thyroid cancer cells showed that the cell cycle inhibitors p21 and p27 were increased by agonist activation or overexpression of PPAR*γ* [12, 21]. Figure 4(c) shows that both p21 and p27 levels were unaffected by PPAR*γ* knockdown in HTh74 ATC cells. 

### 3.3. PPAR*γ* Knockdown Results in Decreased Growth of Flank and Orthotopic Xenograft Thyroid Gland ATC Tumors

**Figure 5:**
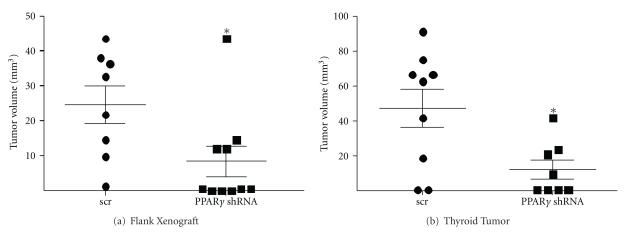
PPAR*γ* knockdown attenuates both flank and thyroid orthotopic tumor growth of HTh74 anaplastic cancer cells. (a) 5 × 10^6^ HTh74 cells stably expressing a scrambled (scr) control (*n* = 8) or PPAR*γ* shRNA (*n* = 10) were injected sc into the right flank of an athymic nude mouse. Tumors were monitored visually and harvested after 8 weeks and measured in 3 dimensions with calipers. Final tumor volume was calculated with the following equation: volume (mm^3^) = L ∗ W ∗ D ∗ 0.523. Mean ± SEM is reported (*, *P* = 0.023, two-tailed *t*-test). (b) 0.5 × 10^6^ HTh74 cells stably expressing a scrambled (scr) control (*n* = 9) or PPAR*γ* shRNA (*n* = 8) were injected into the right thyroid lobe of an athymic nude mouse. Tumors were excised after 8 weeks and measured with calipers as before. The mean tumor volume ± SEM is reported (*, *P* = 0.049, two-tailed *t*-test).

We next examined the effect of PPAR*γ* depletion on *in vivo* tumor growth in both flank and orthotopic xenograft mouse models.  Figure 5(a) shows that the PPAR*γ*-depleted flank tumors were significantly smaller compared with scrambled controls (7.3 ± 3.7 mm^3^ versus 21.4 ± 4.7 mm^3^, *P* = 0.023, two-tailed *t*-test). Injecting tumor cells orthotopically directly into the thyroid represents an *in vivo* site that more closely mimics thyroid cancer in the native environment.  Figure 5(b) shows that PPAR*γ*-depleted ATC tumors were also significantly smaller than scrambled controls in this orthotopic model (12.2 ± 5.4 mm^3^ versus 47.2 ± 11.2 mm^3^, *P* = 0.049, two-tailed *t*-test). Interestingly the eventual size of the orthotopic thyroid tumors was twice that of those grown on the flanks despite being injected with 10-fold fewer cells, suggesting this is a more favorable growth environment for these thyroid cancer cells.

### 3.4. Overexpression of PPAR*γ* in DTC Cells Increases Cell Growth and the Levels of Specific Cyclins

**Figure 6:**
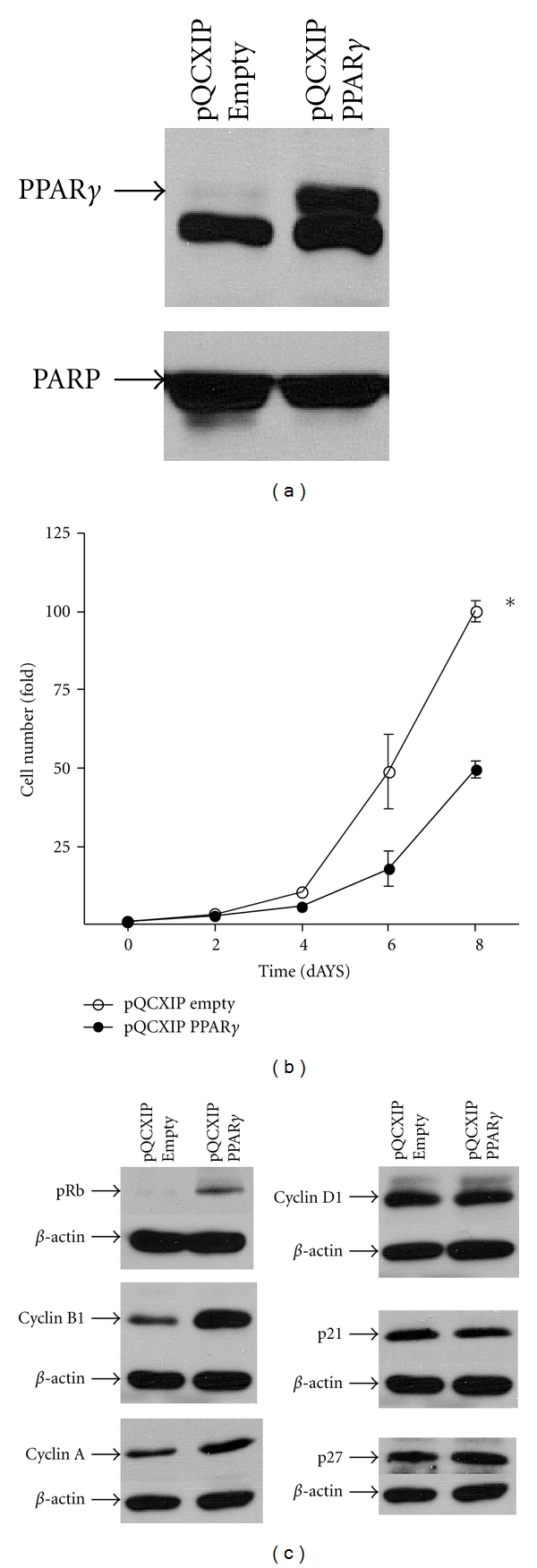
Effect of PPAR*γ* overexpression on the growth characteristics of BCPAP cells. (a) 40 *μ*g of nuclear extract protein from BCPAP cells transfected with either pQCXIP (empty) or pQCXIP-expressing PPAR*γ* was separated on a 10% SDS-PAGE gel, transferred to PVD, probed with PPAR*γ* antibodies (sc-7196), and reprobed with PARP as described in Figure 2(a). (b) Cell growth rate was assessed by counting viable BCPAP cells transfected with pQCXIP (empty) or pQCXIP-expressing PPAR*γ* at 2-day intervals. Data is represented as fold cell number over day 0 (5 expts. ± SEM; *, *P* < 0.001 at day 6, *P* < 0.0001 at day 8 by 2-way ANOVA). (c) 50 *μ*g of total cellular protein from BCPAP cells transfected with pQCXIP (empty) or pQCXIP-expressing PPAR*γ* was separated on a 10% SDS-PAGE gel, transferred to PVD, and probed with antibodies against the corresponding cell cycle protein followed by the appropriate secondary antibody as described in Figure 4. *β*-actin was quantitated as a loading control.

Having shown that depletion of PPAR*γ* in ATC cells resulted in decreased growth both *in vitro* and *in vivo*, we next examined the effect of overexpressing this nuclear receptor in DTC cells.  Figure 6(a) shows successful overexpression of PPAR*γ* in the pQCXIP-PPAR*γ*-transduced BCPAP cells compared with empty vector control.  Figure 6(b) demonstrates a 2-fold increased growth rate in these PPAR*γ*-overexpressing DTC cells. PPAR*γ* overexpression resulted in increased pRb as well as cyclin A and B1 levels whereas cyclin D1, p21, and p27 were again unchanged (Figure 6(c)).

### 3.5. PPAR*γ* Expression Is Associated with Invasion of Thyroid Cancer Cells

**Figure 7:**
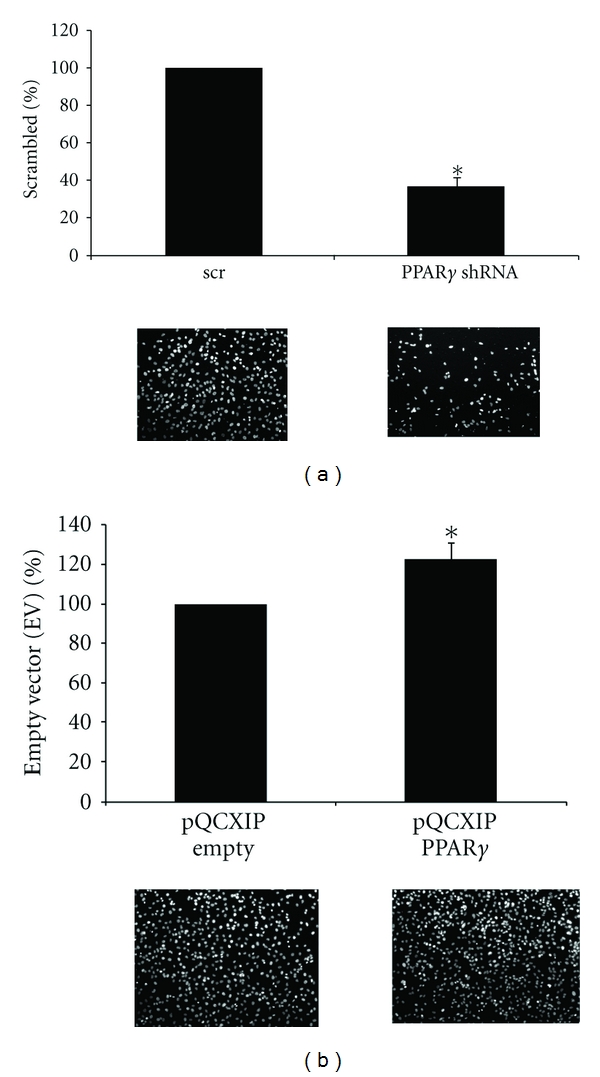
Effect of PPAR*γ* knockdown or overexpression on invasion. (a) HTh74 cells transduced with either scrambled (scr) or PPAR*γ*-specific shRNA were serum starved (0.1% FBS) for 24 h. Cells (2 × 10^5^) were transferred to a Boyden chamber and allowed to invade for 24 h toward medium containing 10% FBS. Cells on the bottom membrane were then stained with DAPI and counted using Metamorph Imaging Software (a representative DAPI-stained field is shown below the graph). Results shown are % inhibition of invading cells per field ± SEM (*, *P* = 0.0015, *n* = 4). (b) Empty vector or PPAR*γ*-overexpressing BCPAP cells were subjected to invasion assays as described in (a) (*, *P* = 0.013, *n* = 3).

Invasion is a tumor feature associated with aggressive behavior [22]. To determine if PPAR*γ* plays a role in the invasive capacity of thyroid cancer cells, we depleted ATC cells of PPAR*γ* and compared this with its overexpression in DTC cells.  Figure 7(a) shows that the invasive capacity of HTh74 ATC cells was inhibited by 40% (60%  ± 0.05%, *P* = 0.0015) by depleting PPAR*γ*. In contrast, Figure 7(b) shows that BCPAP DTC cells overexpressing PPAR*γ* exhibited a 25% increase in invasive capacity (125.5% ± 0.08%, *P* = 0.013) compared with empty vector controls. A representative DAPI-stained field for each experimental situation is shown below the corresponding graph.

## 4. Discussion

In this report, we show that PPAR*γ* protein expression is associated with thyroid cancer type (ATC versus DTC) and not with response to agonist treatment. Abundant expression was found in cells derived from more advanced ATC and was virtually absent in cells from more differentiated TC. Thus PPAR*γ* appears to be a marker for tumor aggressiveness and may play a role in the transition from differentiated (good prognosis) to undifferentiated (very poor prognosis) thyroid cancer. On the other hand, this may simply be an association. Studies of primary thyroid tissue using immunohistochemical analyses generally show low levels of PPAR*γ* in normal thyroid tissue and DTC, suggesting a limited role for this receptor in normal thyroid biology and differentiated cancer function [23–25]. In one of the studies [24], 64% of undifferentiated thyroid cancers had a moderate or high expression of PPAR*γ* whereas only 31% of DTC exhibited any PPAR*γ* expression, lending an important tissue correlation with our cell line data. Two earlier reports which examined PPAR*γ* expression in different thyroid cancer cell lines [11, 12] need to be interpreted with caution as the cell lines used differed from those described here and in fact were shown later to be either redundant or not of thyroid origin [16]. However, another study corroborated our finding of higher PPAR*γ* levels in ATC versus PTC [26], but these cell lines have not been validated by STR profiling [16].

In this paper, we have demonstrated that knocking down PPAR*γ* in the ATC cell line HTh74 not only inhibited *in vitro* cell growth, but also reduced *in vivo* tumor growth in flank and orthotopic xenografts. At least two previous studies utilized an shRNA strategy to knock down PPAR*γ* in thyroid cancer cells. In the first, the investigators did not report what effect PPAR*γ* depletion had on growth only that it reduced the growth inhibitory response to TZD [14]. The second study which included 3 ATC-derived cells [21] demonstrated that knockdown of PPAR*γ* resulted in loss of response to a non-TZD PPAR*γ* agonist and, although not referred to in the text, revealed a 20% decrease in cell growth by depletion of PPAR*γ* alone, which is confirmed by our studies in this report.

A decrease in growth as a result of PPAR*γ* knockdown is somewhat unexpected, given the accumulated data from numerous studies, mainly with PPAR*γ* agonists, that would characterize PPAR*γ* as a tumor suppressor that mediates many antitumorigenic activities such as induction of differentiation, promotion of cell cycle arrest, antiangiogenic effects, and induction of apoptosis [15]. This has been further supported by studies where PPAR*γ* insufficiency leads to an increased incidence of tumors in the liver [27], intestine [28], and even the thyroid [29]. However, there are several studies which would support a role for PPAR*γ* as a tumor promoter, as our data would suggest, especially *in vivo*. Transgenic mice that express a constitutively active PPAR*γ* give rise to greatly exacerbated mammary gland tumor development [30], which supported earlier data that PPAR*γ* agonists may promote tumorigenesis in colon epithelium [8]. Furthermore, a recent report by Reddi et al. [31] demonstrated that forced expression of the PAX8-PPAR*γ* fusion protein, a putative dominant-negative form of PPAR*γ*, in a DTC-derived thyroid cancer cell line (WRO), caused a striking 5-fold reduction in tumor progression in a mouse xenograft model.

Having shown that PPAR*γ* expression levels are higher in ATC cells versus DTC cells and that knocking it down in an ATC cell reduced its growth both in culture and in two *in vivo* xenograft models, we went on to demonstrate that overexpression of PPAR*γ* in a DTC cell line (BCPAP), that lacks detectable PPAR*γ* protein, results in increased cell growth in culture. These combined data would go against the supposed role of PPAR*γ* as a tumor suppressor and suggest that high levels of PPAR*γ*, in the apparent absence of any ligand, may be promoting cancer cell growth. Although to our knowledge no other investigators have done such a direct correlation between PPAR*γ* levels and cell growth, or have manipulated PPAR*γ* expression and assessed its effect on growth, there is evidence in the literature noted earlier that more aggressive tumors [24] or cells derived from such tumors [26] exhibit higher PPAR*γ* levels.

To begin to understand the mechanism whereby PPAR*γ* may be regulating cell growth, we examined cell cycle phase occupancy by flow cytometry and the levels of key cell cycle intermediates that are involved in transit through the cycle. We showed that growth inhibition of ATC cells as a direct consequence of PPAR*γ* knockdown resulted in an increase in G1 phase and a decrease in the S and G2/M phases. This was accompanied by a reduction in phosphorylation of Rb and a concomitant decrease in the critical kinases (cdk1) cyclins (A and B1) but not the D-cyclins. Interestingly, the increase in growth by overexpressing PPAR*γ* in PTC cells was correlated with a corresponding rise in phosphorylated Rb as well as cyclin A and B1 levels but not cyclin D1. Consistent with its role as a key cell cycle regulator, expression of cyclin A is found to be elevated in a variety of tumors [32]. Similarly, the G2/M checkpoint regulators, cdk1 and cyclin B1, have been shown to be expressed at higher levels in more advanced breast cancer lesions [33]. Furthermore, when we examined the levels of two cyclin-dependent kinase inhibitors, p21 and p27, which have been reported to be induced by PPAR*γ* activation in other thyroid cancer cells [12, 21, 34, 35], they were unchanged when PPAR*γ* levels were manipulated. Previous studies on the modulation of the cell cycle by PPAR*γ* have revealed that G1 arrest imposed by PPAR*γ* activation is bypassed in the absence of Rb [36] and is associated with changes in cyclins D3 and E as well as the inhibitor p27. Mechanisms for PPAR*γ* control of the cell cycle involving upregulation of p18 and p21 during adipogenesis [37] or increased p27 but not p21 in pancreatic cancer [38] have also been reported.

Finally, our studies of PPAR*γ* depletion and overexpression show that this receptor increases thyroid cancer cell invasion. *In vitro* invasion assays are used to gauge the migratory potential of a particular cancer cell and increased invasiveness usually signifies a more aggressive phenotype. Our data suggest that PPAR*γ* may be directly contributing to increased invasive capacity. Previous studies have shown that PPAR*γ* activators decrease the invasive capacity of other neoplastic cells. Liu et al. [39] showed that 25 *μ*M rosiglitazone inhibits the invasive properties of human MDA-MB-231 breast cancer cells. More recently, Yang et al. [40] demonstrated that troglitazone (10–30 *μ*M) inhibited migration and invasiveness of a human ovarian carcinoma ES-2 cells but that PPAR*γ* knockdown by siRNA did not reverse this effect underscoring the idea that the PPAR**γ** agonists may reduce cancer cell invasion by a receptor-independent mechanism. This was further supported by the finding that the PPAR*γ* antagonist GW9662, either alone or in combination with troglitazone, does not affect glioma cell invasiveness in a Boyden chamber assay, suggesting that the effects observed are not mediated by PPAR*γ* [41]. Taken together these data strongly suggest that PPAR*γ* agonists inhibit cancer cell invasion through a PPAR*γ*-independent pathway and that as our data suggests PPAR*γ* itself could in fact be promoting invasive capacity. 

In conclusion, our *in vitro* and *in vivo* studies show that PPAR*γ* contributes to more aggressive thyroid cancer properties including faster growth rate and increased invasiveness. Therapies directed at PPAR*γ* expression or a downstream target may lead to novel approaches to treat advanced thyroid cancer.
